# A candidate PTPN5 frameshift variant in a family with a neurodevelopmental disorder supported by a zebrafish loss-of-function model

**DOI:** 10.1016/j.gendis.2026.102159

**Published:** 2026-03-21

**Authors:** Yunlong Meng, Yu Chen, Xingkun Tang, Kangyu Liu, YanLi Ma, Yuanhai Xie, Zilin Zhong, Jianjun Chen

**Affiliations:** a Shanghai Key Laboratory of Anesthesiology and Brain Functional Modulation, Clinical Research Center for Anesthesiology and Perioperative Medicine, Translational Research Institute of Brain and Brain-Like Intelligence, Department of Pediatrics, Shanghai Fourth People’s Hospital, School of Medicine, Tongji University, Shanghai 200434, China; b Institute of Medical Genetics, Department of Big Data in Health Science, School of Public Health and General Medicine, Tongji University School of Medicine, Tongji University, Shanghai 200331, China

Neurodevelopmental disorders are prevalent worldwide and impose a substantial lifelong burden. Despite advances in genomics, many cases still lack a definitive molecular diagnosis. One gene of emerging interest is PTPN5 (protein tyrosine phosphatase, non-receptor type 5), which encodes the striatal-enriched protein tyrosine phosphatase (STEP), a protein widely expressed in the brain and known to regulate synaptic plasticity and signaling.^1^ STEP has also been detected in the retina, suggesting a potential role in visual system biology.^2^ However, current studies on PTPN5 have largely focused on its function in adult animals.^3^ Its role during early developmental stages—especially in neurogenesis and visual evoked behaviors—remains poorly understood. To our knowledge, no clinical reports and no entries in DECIPHER, ClinVar, or GeneMatcher have yet linked intragenic PTPN5 variants to a monogenic human disorder.

We identified a consanguineous Chinese family in which the proband (IV:2) and her affected sister (IV:3) exhibited intellectual disability, speech and learning impairments, and limb anomalies, whereas their parents and two other siblings were unaffected (Fig. 1A; Fig. S1A). Caregivers reported visual concerns in daily life for both affected siblings; however, no formal ophthalmologic examinations (*e.g.*, electroretinography, fundus imaging, optical coherence tomography) were available. Accordingly, the human phenotype is described as neurodevelopmental, with unconfirmed visual symptoms. Whole-exome sequencing was performed on the proband (IV:2), her affected sister (IV:3), and their father (III:2). In this consanguineous family, homozygosity mapping in the two affected siblings revealed several shared runs of homozygosity on multiple chromosomes (Fig. S2A). Within these runs of homozygosity, several rare homozygous coding variants in other genes were identified (Table S1) but were excluded based on zygosity pattern, population allele frequencies, database annotations, and lack of concordance with the clinical phenotype, leaving PTPN5 as the most plausible candidate. A homozygous frameshift variant in *PTPN5* (NM_006906.2:c.1019_1020del) was identified in both affected siblings, while their father was found to be a heterozygous carrier. This variant was absent from the ExAC, 1000 Genomes, and gnomAD databases, suggesting that it is extremely rare. The frameshift variant introduces a premature stop codon, resulting in loss of the STEP protein’s C-terminal catalytic domain (Fig. S1B). Sanger sequencing confirmed co-segregation with disease: affected individuals were homozygous, parents were heterozygous, and unaffected members were either heterozygous or wild-type, consistent with autosomal recessive inheritance (Fig. 1A; Fig. S1C).

**Figure 1:**
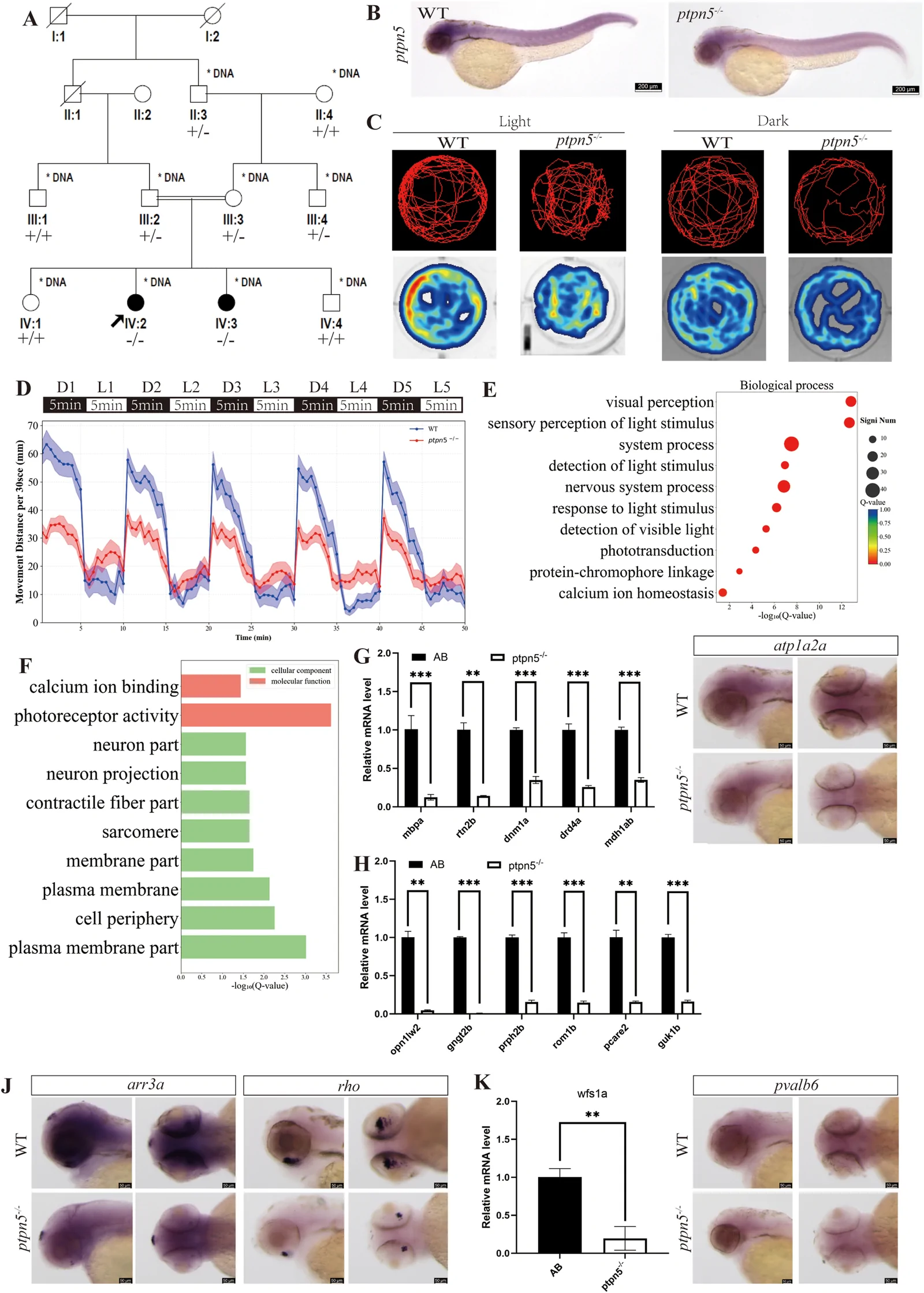
PTPN5 mutations are associated with neurodevelopmental disorders and defective visual motor responses. **(A)** The pedigree of the affected family shows autosomal recessive inheritance of a PTPN5 variant. **(B)** Whole-mount *in situ* hybridization (WISH) confirms expression of ptpn5 in zebrafish larvae; *ptpn5*^*−/−*^ mutants show reduced expression. **(C)** Locomotor trajectories and heatmaps reveal reduced baseline locomotion in *ptpn5*^*−/−*^ larvae under constant light and dark conditions. *n* = 24 larvae per genotype. **(D)** Visual motor response (VMR) assay reveals impaired light–dark transition responses in *ptpn5*^*−/−*^ larvae. Data were presented as mean ± standard error of the mean; *n* = 24 larvae per genotype. **(E, F)** Gene ontology enrichment analysis of differentially expressed genes indicates perturbation of pathways related to visual perception, neurodevelopment, and calcium ion homeostasis. **(G)** Quantitative PCR and WISH validate altered expression of neurodevelopment-associated genes. **(H**–**J)** Validation of visual system genes through quantitative PCR and WISH. **(K)** Reduced expression of calcium homeostasis genes confirmed by quantitative PCR and WISH.

To assess the functional impact of the variant, we performed *in vitro* expression analyses. HeLa cells transfected with EGFP-tagged wild-type or mutant *PTPN5* constructs showed similar reticular cytoplasmic distribution surrounding the nucleus (Fig. S1D). In contrast, quantitative PCR and semi-quantitative reverse-transcription PCR analyses in transfected HEK293T cells revealed that the mutant construct showed significantly reduced EGFP–PTPN5 fusion mRNA and protein levels compared with the wild-type construct (Fig. S1E–S1G). These results indicate that the PTPN5 frameshift variant undergoes nonsense-mediated decay, leading to loss of STEP protein expression.

To investigate the developmental role of PTPN5, we used a zebrafish model. PTPN5/ptpn5 is highly conserved at the sequence level (Fig. S3A). In mammals, PTPN5 is already expressed during prenatal neurodevelopment and increases around birth and into childhood, whereas in zebrafish, ptpn5 is expressed from early embryogenesis, and its expression further increases during larval stages (Fig. S4A and S4B). Consistent with this, in zebrafish, we find that ptpn5 transcripts are detectable from early embryogenesis and become strongly enriched in the developing eyes and central nervous system by 2 days post fertilization (Fig. S5A and S5B), in line with the classical PTP family expression analysis in zebrafish.^4^ Similarly, in mammals, STEP/PTPN5 is expressed not only in the central nervous system but also in the retina.^2^^,^^3^ We generated *ptpn5*-knockout zebrafish via CRISPR/Cas9 gene editing, targeting the site homologous to human Cys340 (Fig. S3A and S5C). This produced a 5-base-pair deletion in zebrafish *ptpn5*, causing a premature stop codon that removed the catalytic domain, mirroring the human variant (Fig. S5D and S5F). Mutant *ptpn5*^*−/−*^ embryos showed dramatically reduced *ptpn5* mRNA expression (Fig. 1B; Fig. S5E). Despite the loss of *ptpn5*, early gross development appeared normal: *ptpn5*^*−/−*^ larvae were morphologically indistinguishable from wild-type siblings in body length, craniofacial structure, and eye size at 2–5 days post-fertilization (Fig. S6A–S6C). Overall neural anatomy (assessed by pan-neuronal and glial markers) was also preserved in mutants (Fig. S6D and S6E).

Although *ptpn5*^*−/−*^ mutants did not display overt anatomical defects, we observed clear neurobehavioral abnormalities. In baseline movement assay under constant light and constant dark conditions, *ptpn5*^*−/−*^ larvae exhibited a significantly reduced total movement distance and active time compared with wild-type larvae (Fig. 1C; Fig. S7A). As baseline locomotion in zebrafish larvae depends on proper neural circuit development, the reduced movement implies defective neurodevelopment. We then evaluated visually evoked locomotor behavior using the visual motor response (VMR) assay under alternating light–dark stimulation. In this assay, alternating periods of light and darkness elicit changes in locomotor activity: wild-type larvae showed rapid bursts of movement at each light-to-dark transition (a “light-off” startle response), which gradually attenuated over repeated cycles as the larvae adapted. In contrast, the *ptpn5*^*−/−*^ larvae had markedly blunted responses to light-off stimuli and failed to exhibit the normal adaptation pattern (Fig. 1D; Fig. S7B). Their activity levels during dark phases remained consistently lower than those of wild-type (Fig. S7C). Together, these behavioral findings support PTPN5 as a candidate gene for neurodevelopmental disorders, with possible visual involvement.

RNA sequencing at 2 days post-fertilization identified 534 differentially expressed genes between *ptpn5*^−/−^ and wild-type larvae (Fig. S8A). Gene ontology analysis revealed that these genes were enriched in relation to neurodevelopment, visual function, and calcium homeostasis (Fig. 1E and F). Genes involved in neuronal differentiation, myelination, and synaptic transmission were significantly changed (Fig. 1G; Fig. S8B). Genes involved in photoreceptor outer segment structure and renewal, phototransduction, and synaptic transmission to bipolar cells were markedly down-regulated (Fig. 1H–J; Fig. S8C and S8D). In addition, transcriptional changes were observed in calcium homeostasis-related genes (Fig. 1K; Fig. S8E), which are closely linked to neural and visual system function. These findings suggest that ptpn5 loss is associated with altered transcriptional programs related to neural signaling and visual processing.

The VMR assay is a circuit-level behavioral readout influenced by tectal processing and modulated by motor circuit function and activity levels. Therefore, neurodevelopmental deficits in *ptpn5*^*−/−*^ larvae may confound visually evoked readouts, so the VMR phenotype cannot be attributed to retina-specific dysfunction. Notably, PTPN5 knockout mice exhibit no visual defects; the zebrafish VMR abnormality may reflect a species-specific neurobehavioral effect of early circuit development rather than a conserved, retina-specific visual deficit.^5^ Accordingly, extrapolation from zebrafish VMR findings to mammalian visual function should be made cautiously. Taken together, these data suggest PTPN5 as a candidate gene for neurodevelopmental disorders, with possible but unconfirmed visual involvement. However, the clinical evidence derives from a single consanguineous family with two affected siblings, and additional unrelated cases will be needed to establish robust genotype–phenotype correlations for PTPN5. Second, in our imaging of *ptpn5*^*−/−*^ larvae at 2–5 days post fertilization, no obvious skeletal abnormalities were noted; however, we did not carry out dedicated skeletal analyses (*e.g.*, cartilage/bone staining or later-stage skeletal imaging), and therefore subtle or delayed skeletal changes in zebrafish cannot be excluded and will require dedicated analysis in future studies. Finally, no retina-specific transcriptomic datasets from STEP/Ptpn5 knockout models are currently available, representing a limitation of the present study and an important direction for future work, together with functional rescue experiments and analyses of patient-derived tissues or cells.

## CRediT authorship contribution statement

**Yunlong Meng:** Writing – original draft, Visualization, Software, Methodology, Investigation, Formal analysis, Data curation, Conceptualization. **Yu Chen:** Writing – review & editing, Validation, Investigation. **Xingkun Tang:** Visualization, Validation, Data curation. **Kangyu Liu:** Validation, Methodology. **YanLi Ma:** Investigation, Formal analysis. **Yuanhai Xie:** Visualization. **Zilin Zhong:** Software, Resources, Project administration, Methodology, Investigation, Conceptualization. **Jianjun Chen:** Writing – review & editing, Supervision, Software, Resources, Project administration, Methodology, Investigation, Funding acquisition, Conceptualization.

## Ethics declaration

All procedures in this study were performed in accordance with ethical standards. Human subject research was approved by the Institutional Review Board of Tongji University School of Medicine (No. 2021tjdx061), with written informed consent obtained from all participants or their legal guardians. All animal experiments were approved by the Animal Ethics Committee of Tongji University (No. TJAA06324B01) and carried out in compliance with institutional guidelines.

## Funding

This work was supported by the Ministry of Science and Technology of China (No. 2021YFA1101300, 2020YFA0112500) and Jiangxi Province’s “Double Thousand Plan” Technology High-end Innovative Talents (China) (No. jxsq2023102228).

## Conflict of interests

The authors declare that they have no known competing financial interests or personal relationships that could have appeared to influence the work reported in this paper.
