# Supplementary material for: A candidate PTPN5 frameshift variant in a family with a neurodevelopmental disorder supported by a zebrafish loss-of-function model
Source: Genes Dis. 2026 Mar 21;14(3):102159. doi: 10.1016/j.gendis.2026.102159 (PMC13718840; doi:10.1016/j.gendis.2026.102159)
Supplement: Supplementary file 1 [file mmc1.docx]

**Supplementary materials and methods**

### **Family recruitment**

We recruited a Chinese family with intellectual disability (ID). The study was approved by the Institutional Review Board of Tongji University School of Medicine (No. 2021tjdx061) (Shanghai, China) and conducted in accordance with the principles of the Declaration of Helsinki. Approximately 5 mL of peripheral venous blood was collected in EDTA-containing vacutainer tubes (Becton Dickinson [BD], Sunnyvale, CA, USA) from available affected individuals and their unaffected family members. Genomic DNA was extracted using a commercial kit (Tiangen Biotech Co., Ltd., Beijing, China). Written informed consent was obtained from all participants or their legal guardians.

### **Whole-exome sequencing (WES) and mutation validation**

Genomic DNA was extracted from peripheral blood samples and subjected to library preparation using the Agilent SureSelect Human All Exon V6 kit, followed by high-throughput sequencing on the Illumina platform (Genesky Bio-Tech Co., Ltd., Shanghai, China). Sequencing reads were aligned to the human reference genome (hg19) using BWA, and variant calling and annotation were performed with the GATK pipeline. Candidate variants were screened for potential pathogenicity and evaluated by co-segregation analysis within the family.

Given the consanguineous background and presumed autosomal-recessive inheritance, identity-by-descent (IBD) and runs-of-homozygosity (ROH) analyses were performed to identify homozygous intervals shared by the two affected siblings. Variant filtering was then restricted to rare homozygous coding variants within shared ROH that were present in both affected individuals and at most heterozygous in unaffected relatives. Population allele frequencies were obtained from gnomAD (v4.1.0 and v2.1.1; overall and East Asian–specific AF), the ExAC v0.3 dataset (overall and East Asian AF), and the 1000 Genomes Project (global and East Asian AF). In-silico predictions (SIFT, PolyPhen-2 and MutationTaster) and reported gene function were further used to prioritise candidate variants (Supplementary Table 1).

To validate the candidate mutation, genomic DNA from available family members was amplified by polymerase chain reaction (PCR) using the following primers for PTPN5 exon 10: Forward primer (PTPN5-EX10F): 5′-CTCCTCCTCATCCTGAGTCCT-3′, Reverse primer (PTPN5-EX10R): 5′-GTCCCTACCTCGTTCATCTCC-3′. PCR products were purified and sequenced using an ABI 3730 Automated Sequencer (Applied Biosystems; Thermo Fisher Scientific, USA). The results were analyzed with Chromas software and compared with the PTPN5 reference transcript (NM_006906) deposited in the NCBI database (http://www.ncbi.nlm.nih.gov/).

### **Plasmid construction**

Total mRNA was extracted from human embryonic stem cells using TRIzol reagent (Tiangen, China). Approximately 1 µg of RNA (OD260/280 ≈ 2.0) was reverse-transcribed into first-strand cDNA using the FastKing One-Step RT-PCR Kit (Tiangen, China). The resulting cDNA was used as a template to amplify the coding sequences (CDS) of both wild-type and mutant PTPN5. The amplified products were cloned into the pEGFP-C1 vector at the XhoI and BamHI restriction sites using the pEASY-Uni® Seamless Cloning and Assembly Kit (TransGen Biotech, China), generating constructs encoding EGFP-tagged wild-type PTPN5 (pEGFP-PTPN5_WT) and mutant PTPN5 (pEGFP-PTPN5_MT). pEGFP-PTPN5_WT and pEGFP-PTPN5_MT express an EGFP–PTPN5 fusion protein under the CMV promoter. EGFP is fused in-frame to PTPN5 and serves as both a fluorescent tag and a reporter of fusion transcript/protein expression. Primers used for amplification are listed in Supplementary Table 2. All constructs were verified by Sanger sequencing to confirm correct insertion and the presence of the intended mutation.

### **Cell culture and transfection**

HeLa and HEK293T cells were cultured in high-glucose Dulbecco’s modified Eagle’s medium (DMEM; Invitrogen, Thermo Fisher Scientific, USA) supplemented with 10% fetal bovine serum (FBS; Invitrogen, Thermo Fisher Scientific, USA) at 37 °C in a humidified incubator with 5% CO₂. Transfections were carried out using Lipo8000™ Transfection Reagent (Beyotime Institute of Biotechnology, China) following the manufacturer’s protocol.

### **Western blot**

HEK293T cells were transfected with 2μg wild-type or mutant plasmids and harvested after 48 h. Cells were lysed on ice using RIPA buffer containing protease inhibitors (P0013B; Beyotime Institute of Biotechnology, China). Lysates were centrifuged at 12,000 rpm for 15 min at 4 °C, and total proteins were collected from the supernatant. Protein concentrations were determined by a bicinchoninic acid (BCA) assay (Beyotime Institute of Biotechnology, China). Equal amounts of protein were denatured in SDS loading buffer (Beyotime Institute of Biotechnology, China), separated by 8% SDS–PAGE, and transferred to polyvinylidene fluoride membranes. Membranes were blocked with 5% non-fat milk at room temperature for 1 h and then washed three times with TBST. EGFP-PTPN5 recombinant proteins were detected using mouse monoclonal anti-EGFP (1:1000) and anti-ACTB (1:1000) antibodies (Sangon Biotech Co., Ltd., China) at 4 °C overnight or at 25 °C for 2 h, followed by incubation with goat anti-mouse IgG secondary antibody (1:5000; Sangon Biotech Co., Ltd., China). Signals were visualized with an enhanced chemiluminescence substrate (Bio-Rad Laboratories, USA) using a Tanon Imaging System (Tanon Science & Technology Co., Ltd., China). Band intensities were quantified with ImageJ (version 1.54k; NIH, USA), and relative protein expression was normalized to ACTB.

### **Immunofluorescence**

HeLa cells (3 × 10⁴ per well) were seeded on coverslips in 12-well plates in DMEM containing 10% FBS, transfected with 1 µg of plasmid DNA per well, and after 36 h were washed three times with PBS and fixed with 4% paraformaldehyde for 20 min, followed by three additional PBS washes. Nuclei were counterstained with DAPI (0.5 µg/mL) for 1 min and washed three times with PBS. The subcellular localization of wild-type and mutant PTPN5 proteins was examined by confocal fluorescence microscopy at 40× magnification.

### **Zebrafish husbandry**

Wild-type AB zebrafish (Danio rerio) and the transgenic lines *Tg(elavl3:YC2)* and *Tg(olig2:mCherry)* were used in this study. All fish strains were obtained from the China Zebrafish Resource Center (Wuhan, China). Zebrafish were maintained in a recirculating aquaculture system at 28.5 °C under a 14 h light/10 h dark cycle with water quality control according to standard protocols. Fish were fed freshly hatched brine shrimp twice daily. Natural mating was used to obtain fertilized eggs, which were collected and cultured in E3 medium. Embryos at different developmental stages were harvested for RNA analysis and whole-mount in situ hybridization. At the end of the experiments, zebrafish were humanely euthanized. All experimental procedures were approved by the Animal Ethics Committee of Tongji University (No. TJAA06324B01) and performed in accordance with institutional guidelines.

### **Generation of ptpn5 knockout zebrafish**

Based on the human pathogenic mutation site, guide RNAs (gRNAs) targeting the zebrafish ptpn5 gene were designed using CHOPCHOP ([http://chopchop.cbu.uib.no/#](http://chopchop.cbu.uib.no/)). Single-guide RNAs (sgRNAs) were synthesized in vitro and incubated with Cas9-EGFP protein at a molar ratio of 1.2:1 at 37 °C for 10 min to form ribonucleoprotein (RNP) complexes. The RNPs were co-injected into one-cell stage embryos, and embryos with strong EGFP fluorescence were selected for further culture at 3–6 hours post fertilization (hpf). At 1 day post fertilization (dpf), dishes containing embryos with mutations (F0) were identified and raised to adulthood. Adult F0 fish were crossed with AB wild-type zebrafish to obtain heterozygous carriers, and Sanger sequencing confirmed a 5 bp deletion in the ptpn5 locus. Stable mutant lines (F2) were established through self-crossing of F1 heterozygotes. The effects of the mutation were validated by quantitative reverse transcription PCR (qPCR) and whole-mount in situ hybridization (WISH). Sequences of the sgRNAs and genotyping primers are provided in Supplementary Table 3.

### **Morphological and neurodevelopmental analysis**

Morphological differences between wild-type (WT) and ptpn5⁻/⁻ embryos were assessed by measuring body length and interocular distance. Images were captured using a stereomicroscope (Leica M205 FCA, Germany), and measurements were performed with ImageJ software (version 1.54k; NIH, USA). At least 25 embryos were analyzed per group. To assess neurodevelopment, *ptpn5^⁻/⁻^* fish were intercrossed with *Tg(elavl3:YC2)* and *Tg(olig2:mCherry)* lines to generate homozygous mutants carrying the heterozygous transgenic background. The distribution of neurons and oligodendrocytes was examined under a Leica M205 FCA stereomicroscope.

### **Behavioral analysis**

Behavioral assays were performed at 6 days post-fertilization (dpf) to evaluate baseline movement and the visual motor response (VMR). Larvae were transferred to 48-well plates with one larva per well (24 WT and 24 *ptpn5^⁻/⁻^* per assay). The recording chamber was illuminated from below with infrared (IR) LEDs, enabling the camera positioned above the chamber to track fish movements even in darkness. As zebrafish larvae cannot detect IR light, this constant illumination did not interfere with behavior. White LEDs placed beneath the chamber provided visible light stimuli and were controlled independently of the IR system. For baseline movement, larvae were acclimated for 1.5 h under alternating light/dark conditions to minimize handling stress, followed by 20 min of continuous video recording. For the VMR assay, larvae were acclimated under light for 1.5 h and subsequently exposed to five consecutive cycles of 5-min light and 5-min dark periods.

Behavioral recordings were acquired using a high-resolution camera and analyzed with a custom Python-based automated tracking pipeline that extracted larval centroids on a frame-by-frame basis and computed locomotor parameters. Fish position in each frame was converted into x–y coordinates, and locomotor metrics (distance moved and time spent active) were calculated from frame-to-frame displacement using a pixel-to-distance conversion factor calibrated with objects of known length in the recording field. The experimenter was aware of genotype during acquisition; however, all analyses were performed using predefined, automated pipelines applied identically to all groups, which helped to minimize subjective bias.

### **Transcriptome sequencing (RNA-seq)**

At 2 dpf, heads from WT and *ptpn5^⁻/⁻^* larvae were collected (approximately 120 larvae per sample), with three biological replicates per group. Total RNA was extracted as described above and subsequently submitted to Sangon Biotech (Shanghai, China) for quality control, library preparation, sequencing, and bioinformatic analysis. Briefly, RNA integrity was evaluated by NanoPhotometer, Qubit fluorometer, and agarose gel electrophoresis. High-quality RNA was used for cDNA library construction and paired-end sequencing on the Illumina NovaSeq platform. Data processing and analysis were performed by Sangon Biotech, including adaptor and low-quality read removal with Trimmomatic, alignment to the zebrafish reference genome with HISAT2, quantification of gene expression using StringTie, and normalization to transcripts per million (TPM). Differentially expressed genes (DEGs) were identified with DESeq2 (q < 0.05, |log₂FC| ≥ 1). Functional enrichment analyses of DEGs were conducted using Gene Ontology (GO) and Kyoto Encyclopedia of Genes and Genomes (KEGG) databases, with significance defined as FDR < 0.05. Some candidate genes were verified by qPCR and WISH.

### **Gene mRNA analysis**

Total RNA was extracted from HeLa cells (36 h post-transfection with wild-type or mutant plasmids) and from zebrafish at different developmental stages or heads at 2 dpf using TRIzol reagent (Tiangen, Beijing, China). cDNA synthesis was performed as described above. qPCR was carried out using SYBR Green Master Mix (Tiangen, Beijing, China) on a LightCycler® real-time PCR system (Roche, Switzerland). β-actin was used as the housekeeping gene for normalization, and relative expression levels were calculated using the 2^^−ΔΔCT^ method. For cell experiments (HEK293T), gene expression was additionally assessed by Semi-quantitative RT-PCR (semi-qRT-PCR). PCR products were separated on agarose gels. Primer sequences are listed in Supplementary Table 2 and 3.

### **Whole-mount in situ hybridization (WISH)**

Antisense probes were synthesized by in vitro transcription of target gene fragments with the T7 DIG RNA Labeling Kit (Roche, Switzerland). The procedure followed previously described protocols.^1^ After staining, embryos were stored in 100% glycerol, and hybridization signals were imaged using a stereomicroscope (LEICA M205 FCA, Germany). PCR primers used for probe synthesis are listed in 2.

### **Analysis of public transcriptomic datasets**

Human PTPN5 developmental expression was analysed using RNA-seq data from the BrainSpan: Atlas of the Developing Human Brain (https://www.brainspan.org/), which forms part of the prenatal human brain atlas described by Miller et al. (Nature, 2014)^2,3^. Gene-level RPKM values and sample metadata were downloaded and stored in a tabular file. All preprocessing was performed in Python using pandas (v2.3.3) and Matplotlib (v3.10.6). For each donor-age category (donor_age), PTPN5 log₂(RPKM) values were extracted across all sampled brain regions, and the maximum log₂(RPKM) within that donor-age category was used as a summary measure of global brain expression at that age. The resulting maximum log₂(RPKM) values were plotted as a line graph against donor age using Matplotlib, thereby summarising developmental changes in PTPN5 expression across human prenatal and postnatal brain development.

Zebrafish ptpn5 expression dynamics were extracted from the Zebrahub single-embryo scRNA-seq time-course atlas (https://zebrahub.sf.czbiohub.org/), which is described in Lange et al. (Cell, 2024)4,5. The processed atlas file (zf_atlas_full_v4_release_zebrahub.h5ad) was loaded into Python (v3.12) as an AnnData object using Scanpy (v1.11.5). Cells were stratified by developmental timepoint using the timepoint metadata column (10hpf, 12hpf, 14hpf, 16hpf, 19hpf, 24hpf, 2dpf, 3dpf, 5dpf, 10dpf). For each timepoint, we extracted the normalized expression values of ptpn5 from the expression matrix and computed the arithmetic mean across all cells. The resulting mean expression values (on the original, non–log-transformed scale) were visualized in Matplotlib (v3.10.6) as a line plot with developmental timepoint on the x-axis and mean ptpn5 expression on the y-axis, using a logarithmic (base 10) y-axis scale.

### ****Statistical analysis****

All statistical analyses were performed using GraphPad Prism (version 10.5.0). Data from molecular and cellular experiments (including Western blot, qPCR, and semi-qRT-PCR are presented as mean ± standard deviation (SD). Zebrafish behavioral assays (baseline movement and VMR) are presented as mean ± standard error of the mean (SEM). Homogeneity of variance between groups was assessed using an F-test. When variances were not significantly different (P > 0.05), comparisons between two groups (e.g., WT vs. *ptpn5^⁻/⁻^*) were performed using unpaired two-tailed Student’s t-tests (equal variance). When variances differed significantly (P < 0.05), Welch’s t-tests (unequal variance) were applied. A *P* value < 0.05 was considered statistically significant. All experiments were independently repeated at least three times unless otherwise specified.

**Supplementary references**

1.	Cao Z, Meng Y, Gong F, et al. Calcineurin controls proximodistal blastema polarity in zebrafish fin regeneration. *Proc Natl Acad Sci U S A*. Jan 12 2021;118(2)doi:10.1073/pnas.2009539118

2.	BrainSpan. Atlas of the Developing Human Brain. Allen Institute for Brain Science. 2025. <https://www.brainspan.org/>

3.	Miller JA, Ding SL, Sunkin SM, et al. Transcriptional landscape of the prenatal human brain. *Nature*. Apr 10 2014;508(7495):199–206. doi:10.1038/nature13185

4.	Lange M, Granados A, VijayKumar S, et al. A multimodal zebrafish developmental atlas reveals the state-transition dynamics of late-vertebrate pluripotent axial progenitors. *Cell*. Nov 14 2024;187(23):6742–6759 e17. doi:10.1016/j.cell.2024.09.047

5.	Zebrahub. Multimodal Zebrafish Developmental Atlas. CZ Biohub San Francisco. 2025. <https://zebrahub.sf.czbiohub.org/>

**Supplementary figures**

**Figure S1**

**Figure S1. Characterization of the PTPN5 Mutation in Affected Individuals.** **(A)** Photographs of the limbs of the proband, an affected sibling, and an unaffected sibling. The proband (IV:2) and her affected sibling (IV:3) have short and thick distal phalanges, mild clinodactyly, and hypertrophic and blunted digits. The toes (especially the big toe) are shortened, broadened, and bilaterally symmetrical. The unaffected sibling (IV:1) has normal-appearing hands and feet. **(B)** Schematic representation of the PTPN5 protein domains. The c.1019_1020del mutation introduces a premature stop codon (p.Cys340SerfsTer7), resulting in the loss of the catalytic domain.**(C)** Sanger sequencing chromatograms showing the wild-type, heterozygous, and homozygous c.1019_1020del variants in PTPN5. **(D)** Immunofluorescence analysis of EGFP-tagged PTPN5-WT and PTPN5-MT proteins in cells. Both localize to perinuclear reticular structures (green: EGFP; blue: DAPI). **(E)** qPCR shows significantly reduced transcript levels of PTPN5-MT compared to WT. **(F)** Semi-quantitative PCR confirms decreased expression of PTPN5-MT. **(G)** Western blot analysis of EGFP-tagged PTPN5-WT and -MT proteins reveal markedly reduced mutant protein levels. β-actin serves as a loading control.

**Figure S2**

**Figure S2. Homozygosity mapping in the affected siblings. (A) Circos plots showing genome-wide runs of homozygosity (ROH) in IV:2 (left) and IV:3 (right). Green segments indicate ROH, and arrows mark shared ROH on chromosomes 1, 4, 11, 13, and 14, which were used for variant prioritization.**

**Figure S3**

**Figure S3. Conservation analysis of PTPN5 protein.** (A) Multiple sequence alignment of PTPN5 across species shows high conservation of p.Cys340, the KIM domain, and the catalytic domain.

**Figure S4**

**Figure S4. Developmental expression of PTPN5/ptpn5 in human and zebrafish. (A)** PTPN5 expression across human brain development based on the BrainSpan RNA-seq developmental transcriptome. For each donor, the maximum log2 RPKM value of PTPN5 across all sampled brain regions was calculated and plotted against donor age (post-conception weeks, pcw, for prenatal samples; years for postnatal samples). **(B)** ptpn5 expression across zebrafish development in the Zebrahub single-cell RNA-seq time-course atlas. For each developmental timepoint, the mean log1p-transformed expression of ptpn5 was calculated across all cells and plotted on a logarithmic y-axis.

**Figure S5**

**Figure. S5. Temporal and spatial expression of ptpn5 during zebrafish embryonic development and construction of KO model.** **(A)** Whole-mount in situ hybridization revealed maternal expression of ptpn5 at the 8-cell stage, with progressive upregulation from 24 to 72 hpf, predominantly localized to the central nervous system. **(B)** qPCR analysis showed stage-dependent increases in ptpn5 mRNA levels during early development. **(C)** Schematic of zebrafish ptpn5 gene with the sgRNA target site indicated. **(D)** Sanger sequencing confirmed a 5-bp deletion generating a premature stop codon. **(E)** qPCR validation demonstrated significantly reduced ptpn5 transcript levels in mutants. **(F)** Protein schematic illustrating loss of the catalytic domain in mutant ptpn5 compared with wild type. Data are presented as mean ± SD; *P < 0.05, **P < 0.01, ***P < 0.001; ns, not significant.

**Figure S6**

**Figure. S6. Early developmental morphology and neural marker expression in *ptpn5^⁻/⁻^* zebrafish embryos.** **(A)** Representative bright-field images of WT and *ptpn5^⁻/⁻^* embryos at 2-5 dpf show no obvious gross morphological abnormalities. Scale bar: 500 μm. **(B–C)** Quantification of body length (B) and interorbital distance (C) revealed no significant differences between WT and *ptpn5^⁻/⁻^* embryos in 2-5dpf. **(D)** Whole-mount fluorescence in situ hybridization of *elavl3:YC2* transgenic embryos at 2 dpf. *ptpn5^⁻/⁻^* embryos exhibited normal overall neuronal distribution compared with WT controls. Scale bar: 250 μm. **(E)** Confocal imaging of *olig2:mCherry* transgenic embryos at 4 dpf. Motor neuron distribution and axonal projections appeared grossly intact in *ptpn5^⁻/⁻^* embryos relative to WT. Scale bar: 50 μm.

**Figure S7**

**Figure S7. Effects of ptpn5 deficiency on baseline locomotor and visuomotor responses in zebrafish.** (A) Baseline locomotor activity under constant light or dark conditions. Compared to WT, *ptpn5^⁻/⁻^* mutants exhibited significantly reduced total distance and activity time in both light and dark conditions. (B) Quantification of VMR, including distance and time spent active (≥2 mm/s), during each light period. Compared to wild-type, the mutant showed a significant decrease in distance and time spent active during all dark phases, with no significant differences during the light phase. (C) Total distance during the VMR experiment and the cumulative distance of the light and dark phases. Although a trend toward decrease was observed in the mutant, no significant differences were observed overall. The mutant showed a significant decrease in distance during the dark phase, with no differences during the light phase. Data are presented as mean ± SEM; n = 24 larvae per genotype; *P < 0.05, **P < 0.01, ***P < 0.001; ns, not significant.

**Figure S8**

**Figure S8. Transcriptomic alterations in *ptpn5^⁻/⁻^* embryos highlight dysregulation of calcium homeostasis, neurodevelopment, and visual system pathways.** (A) Volcano plot of RNA-seq data from 2 dpf head tissues showing 199 upregulated and 335 downregulated genes in *ptpn5^⁻/⁻^*versus WT larvae. (B) Heatmap of differentially expressed genes involved in neurogenesis and neuronal development. (C) Heatmap of genes related to photoreceptor outer segment formation, phototransduction, and photoreceptor-to-bipolar cell synaptic transmission. (D) WISH revealed reduced retinal expression of arl3l2 and gnb3b in *ptpn5^⁻/⁻^* larvae compared with WT. Scale bars: 50 μm. (E) Heatmap showing alterations of genes involved in calcium homeostasis.

**Supplementary tables**

**Supplementary Table 1. The information of the candidate variants.**

Note: Chr, chromosome; AF, allele frequency; T, tolerance; B, benign; N, neurtral; D, deleterious; P, possibly damaging.

**Supplementary Table 2.** List of primer sequences employed for PTPN5 mutation verification, generation of EGFP-PTPN5 (WT/MT) fusion constructs, and quantitative analysis of PTPN5 expression in cultured cells.

| PTPN5-EX10F | ctcctcctcatcctgagtcct |
| --- | --- |
| PTPN5-EX10R | gtccctacCTCGTTCATCTCC |
| pEGFP-C PTPN5 FP | GTCCGGACTCAGATCTCGAGGCATGAATTATGAGGGAGCCAGG |
| PTPN5_CDS RP | TTCTGGGGACTGGTGGGA |
| pEGFP-C PTPN5 RP | GTTATCTAGATCCGGTGGATCCTCATTCTGGGGACTGGTGGGA |
| PTPN5 c.947_948del RP | CTGGTGAGGTCAGACACTCTGCTGTG |
| PTPN5 c.947_948del FP | CACAGCAGAGTGTCTGACCTCACCAG |
| pEGFP RP | CTGATTATGATCAGTTATCTAGATCCGGTG |
| EGFP_qPCR FP | CAAGGAGGACGGCAACATC |
| EGFP_qPCR RP | GTTCTGCTGGTAGTGGTCGG |
| hβ_ACTIN qPCR FP | CCCAGAGCAAGAGAGGCAT |
| hβ_ACTIN qPCR RP | AAACATGATCTGGGTCATCTTC |

**Supplementary Table 3.** List of primer sequences employed in zebrafish knockout (KO) generation, quantitative PCR (qPCR), and whole-mount in situ hybridization (WISH) experiments.

| ptpn5 sgRNA | taatacgactcactataggAGTGTGCTTAAAAGCTAAGGgttttagagctagaaata |
| --- | --- |
| gRNA RP | AGCACCGACTCGGTGCCACT |
| ptpn5 D FP | ggggtggtgaggaagaatcg |
| ptpn5 D RP | tgggatggaagcaaggtcag |
| ptpn5 qPCR FP | gcaggaaaggagaggctctaatg |
| ptpn5 qPCR RP | gggagtctcatagaactcggc |
| gfap qPCR FP | AGCTACATCGAGAAGGTGCG |
| gfap qPCR RP | CAGGGCAGTCTCGTCTTGAA |
| olig2 qPCR FP | GTCTCGCATTCCTCTCACCC |
| olig2 qPCR RP | GCTCATACCGGGAACGTGAG |
| sox10 qPCR FP | CAGAGTCACAGCCCTCCAAC |
| sox10 qPCR RP | GGCTGATTTCGCCAATGTCC |
| gap43 qPCR FP | AATAGACAAACCAGACGCTGC |
| gap43 qPCR RP | CGAACATAAAGCAGGCTGTCG |
| neurog1 qPCR FP | CCAGCCCACCAATAAGGTTATCA |
| neurog1 qPCR RP | ATCCGTGTGCGAAAAGGAGT |
| sox9b qPCR FP | ACCCCACATTCAGCTACAGC |
| sox9b qPCR RP | CACTGTTTGCTGTGGAGTGC |
| wfs1a qPCR FP | CAAGCATTGCCATTTCCGCT |
| wfs1a qPCR RP | CGGGGAAGTTTACCGGAGTC |
| mbpa qPCR FP | AACTATTGGGCCTGGCTTCC |
| mbpa qPCR RP | ATCGGCTTTCTCCCAGGTTG |
| rtn2b qPCR FP | CCTCCTGCCCCTGCATTAAA |
| rtn2b qPCR RP | AGCCCCAAACCATTGAACCT |
| dnm1a qPCR FP | AGAACATCCACGGCATCAGG |
| dnm1a qPCR RP | TTTGCGAATGGTGGAGGTGA |
| drd4a qPCR FP | CTCTGCGCCATCAGCATAGA |
| drd4a qPCR RP | GTTGATGCCGAACATCACGG |
| mdh1ab qPCR FP | GGAGAGCAGCTGTCATTCGT |
| mdh1ab qPCR RP | CATCTCCCTTTGCAATGCCG |
| opn1lw2 qPCR FP | CTTCATGAACCGACAGTTCCG |
| opn1lw2 qPCR RP | GTTCAACAGGAGCTATAAATCACG |
| gngt2b qPCR FP | CGACAATCCACAGTCAGGATG |
| gngt2b qPCR RP | AGCAGTGTTCACTTCCACCT |
| prph2b qPCR FP | TACAGCTACGACCACCACAC |
| prph2b qPCR RP | CAGTCACGACAGCCTGCATAA |
| rom1b qPCR FP | GCTGGTGGCCATGATCCTAA |
| rom1b qPCR RP | CTGGTGTGTCTGTGTCCTTG |
| pcare2 qPCR FP | GCCAAGGCCAGCTATCTGAA |
| pcare2 qPCR RP | AATGGCGGTGCTTTGGTTTC |
| guk1b qPCR FP | CATCTGACCTTCACAGACACGA |
| guk1b qPCR RP | TGTGAGAGACGCTGAAACCA |
| ptpn5 prob FP | tcctgctctacacactgtctcaa |
| ptpn5 prob RP | acgtaatacgactcactatagggactgcagtggacgatgacag |
| pvalb6 prob FP | GAGCAGCATCCTCAACCATG |
| pvalb6 prob RP | taatacgactcactatagggGTGTGTGATGGGGTGATGT |
| atp1a2a prob FP | TCAGCATGGCTTACGGACAG |
| atp1a2a prob RP | taatacgactcactatagggCCCAGCCTCCTGGATTTCTC |
| arr3a prob FP | CCCTGCTCTGTTACGCTTCA |
| arr3a prob RP | taatacgactcactatagggTCTTTAAGCCTGCCGTCCAG |
| rho prob FP | CAACTTCCGTTTCGGGGAGA |
| rho prob RP | taatacgactcactatagggCAGGGTGGTGATCATGCAGT |
| arl3l2 prob FP | GGTTTGAGGAAACAGGACTGGAG |
| arl3l2 prob RP | taatacgactcactataggGTGCTGTGTGCTCTGAGTGTAA |
| gnb3b prob FP | CGATTGAAGAGCTGTTTTGTGGA |
| gnb3b prob RP | taatacgactcactatagggCTGGAAGGTCTGCTTGCACT |
